# Circadian Rhythms, Regular Exercise, and Cognitive Performance in Morning-Trained Dancers

**DOI:** 10.3390/clockssleep7010007

**Published:** 2025-02-18

**Authors:** Mariana Marchesano, Alejandra Carboni, Bettina Tassino, Ana Silva

**Affiliations:** 1 Grupo Cronobiología, Comisión Sectorial de Investigación Científica, Universidad de la República, Montevideo 11400, Uruguay; mmarchesano@fcien.edu.uy (M.M.); tassino@fcien.edu.uy (B.T.); 2 Facultad de Psicología, Universidad de la República, Montevideo 11200, Uruguay; alejandra.carboni@psico.edu.uy; 3 Sección Etología, Facultad de Ciencias, Universidad de la República, Montevideo 11400, Uruguay; 4 Laboratorio de Neurociencias, Facultad de Ciencias, Universidad de la República, Montevideo 11400, Uruguay

**Keywords:** attention, dance, circadian challenge, young adults, moderate physical activity

## Abstract

Time-of-day and individual circadian variability influence cognitive performance, with later chronotypes being most compromised earlier in the day. On the other hand, moderate-intensity exercise has been shown to enhance cognitive function. We sought to evaluate the interplay among circadian rhythms, exercise, and cognitive performance in 22 students from the Uruguayan National Dance School, a population previously characterized as late chronotypes, attending a demanding morning schedule. We assessed sleep habits and physical activity patterns using self-report questionnaires and actigraphy. Before and after morning training, participants completed a psychomotor vigilance task (PVT) and a visual Stroop task (congruent and incongruent). The reaction speeds were lower early in the morning than at noon for all these tasks. We also found (1) a positive correlation between weekend sleep duration and PVT performance before training but not after; (2) a negative correlation between individual circadian phase and Stroop performance for both congruent and incongruent conditions after training but not before; and (3) a better Stroop performance after training for both congruent and incongruent conditions in dancers who engaged longer moderate-intensity exercise during training. Our findings suggest that regular morning training might help mitigate cognitive impairments experienced by dancers with later chronotypes in challenging morning scenarios.

## 1. Introduction

Knowledge of human biological rhythms has contributed to improving several sectors of modern life, including health, education, and sports [1,2,3]. From a biological perspective, adjusting to environmental rhythmicity enables organisms to anticipate periodic events such as daily changes in temperature or light or food availability, as well as to efficiently regulate behavioral and metabolic processes [4]. Many physiological functions show endogenous ~24 h oscillations strongly entrained by the light–dark cycle [5], but circadian rhythms are also sensitive to nonphotic inputs such as mealtimes and social stimuli [6]. Exercise influences the human circadian system, with the effects depending on when during the day a person exercises [7,8].

Both cognitive and motor performance exhibit circadian variations [3,9,10]. Attentional levels are generally low in the early morning and reach their highest levels around midafternoon [11], while most components of exercise performance peak in the early evening [12]. Individual differences in the entrainment of circadian clocks to the rhythmic environment are often referred to as “chronotypes”, with early “larks” and late “owls” at the extremes of a normal distribution [13]. Previous studies revealed that peak performance times vary among individuals depending on their chronotype [11,14] with late chronotypes demonstrating poorer cognitive and motor performance in the morning compared with early chronotypes [15]. On the other hand, morning exercise has been found to help individuals with late chronotypes overcome morning challenges by advancing their circadian phase [16]. Furthermore, a comprehensive intervention that included targeted light exposure, meals, and caffeine along with exercise in the morning reported improvements in cognitive and physical performance during ‘suboptimal’ morning hours [17].

Physical activity enhances cognitive function, with different effects depending on the duration, frequency, intensity, and type of exercise [18]. The effect of exercise also depends on which specific cognitive skill is evaluated and what time of day it is tested [19,20,21]. Precisely, there is strong support for positive effects of moderate-intensity exercise on cognition [22]. These include improvements in reaction speed induced by acute physical exercise in male trained athletes [23] and young sport students [24] and improvements in attention in response to moderate-intensity exercise in young female physical education students [25]. Professional dancers perform at moderate to vigorous intensities, requiring not only physical strength and endurance but a high level of visual attention for precision, group coordination, and inhibitory control [26,27]. While there is abundant research on sports and cognition across various age groups, studies on dance as a potential cognitive enhancer have been largely focused on elderly populations [28,29]. Moreover, most research on exercise and cognition has been conducted in controlled laboratory settings, often using bicycle or treadmill ergometry [30]. However, less is known about the effect of dance on cognition in real-world contexts, particularly from a chronobiological perspective [31].

Here, we examined the interplay among circadian parameters, physical activity features, and attention in young adult subjects in the real-life setting of the Uruguayan National Dance School (ENFAS) [32,33,34]. Although most of these dance students had shown late chronotypes [35], their 4 h daily training began in the early morning (08:30 h). This situation prompted us to examine how movement and circadian parameters interacted in a regular, challenging schedule for late-chronotype dancers and how they were associated with attentional attributes.

We expected to observe differences in cognitive performance before and after training, with faster responses following the 4 h morning practice compared with the early morning measurement. Additionally, we predicted that the later the individual’s circadian phase was, the lower their attentional levels would be. We also expected to find an association between time spent in moderate-intensity exercise during training and improved performance.

## 2. Results

### 2.1. Circadian and Exercise Characterization

We characterized 22 young dancers, mostly female, who regularly trained during the morning (08:30–12:30, Monday to Friday) (Table 1). The majority (73%, 7 participants excluded because of alarm use on free days) were late chronotypes (MCTQ, Midsleep point on free days corrected for sleep debt on workdays, MSFsc above 05:00), with very high social disruption (MCTQ, SJL > 2 h) and intermediate circadian preference (MEQ, between 41 and 59). Regarding characterization by objective accelerometry, we first assessed the midpoint of the least active five hours on free days (L5cf) as a proxy of the individual circadian phase, which was above 04:30 in average (Table 1). In addition, we found that participants exhibited moderate interdaily stability (IS), suggesting moderate consistency in their daily activity patterns; low intradaily variability (IV), reflecting low fragmentation of the rest–activity pattern; and a high relative amplitude (RA), indicating a healthy balance between the most and least active times of day.

According to accelerometer data (*n* = 21), dancers slept the recommended number of hours for their age (>7 h) in the weekly average. We found significant differences in sleep duration between training days and free days as estimated by MCTQ but not by accelerometry (Table 2). We found significant differences in sleep onset and sleep-end between training days and free days as estimated by both MCTQ and accelerometry.

Comparing self-reported (MCTQ) and objective measures (actimetry), we found a correlation in sleep duration on both workdays (R2 = 0.48, *p* = 0.003) and free days (R2 = 0.32, *p* = 0.0016) and a correlation in sleep onset on training days (R2 = 0.32, *p* = 0.014). Interestingly, self-report SD was shorter than actimetric SD during training days and longer during free days (mean difference: 1.1, *p* = 5.23 × 10^−5^, d = 0.6; mean difference: 0.94, *p* = 0.018, d = 1.01). We found a correlation between the circadian phase, as estimated by L5c on free days, and chronotype, as estimated by MSFsc (R2 = 0.29, *p* = 0.023). However, L5c and the midsleep point did not correlate on either training days or free days (*p* > 0.05). On the other hand, MEQ score did not correlate either with MSFsc or L5c on free days (*p* > 0.05).

We also used accelerometry to assess exercise features. Participants exhibited high levels of moderate physical activity (MPA) during training hours, with an average of 167 (23) mg and 48 (22) mins per session. Additionally, they engaged in moderate to vigorous physical activity (MVPA) for 168 (69) min per day. Interestingly, we detected a bimodal distribution in the time spent in MPA during the training hours. The most active dancers spent on average 62.4 (7) min in MPA during the 4 h training, while the less active group spent 20.6 (12.9) min (*p* < 0.001, d = 2.29). As expected, active dancers also showed a higher RA on training days (mean difference: 0.12, *p* = 0.002, d = 2.04), reflecting more robust activity circadian rhythms, a less negative intensity gradient (mean difference: 0.47, *p* < 0.001, d = 3.62), and a lower gradient intercept (mean difference: −1.6, *p* < 0.001, d = 3.63), which in turn reflected more time spread across the intensity range. We did not find significant differences with respect to IV, IS, or SJL between the active movers and the light movers (*p* > 0.05).

### 2.2. Cognitive Performance

**Figure 1:**
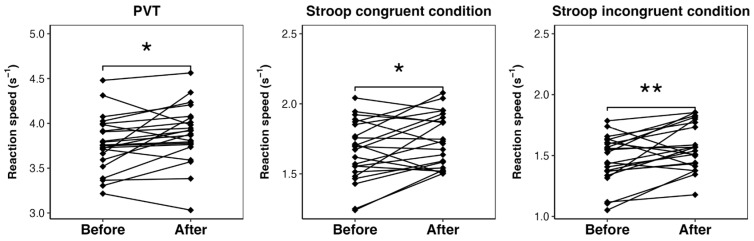
Individual variation in performance before and after morning training sessions. Paired *t*-tests were applied. Significance is presented as * = *p* < 0.05, ** = *p* < 0.01.

Regarding attentional performance, consistently with circadian variation, we found significant differences in both PVT and Stroop tasks before and after training, with faster reaction at 12:30 (Figure 1). More pronounced effects were observed in the task requiring greater cognitive effort (Stroop IC) (Table 3).

**Figure 2:**
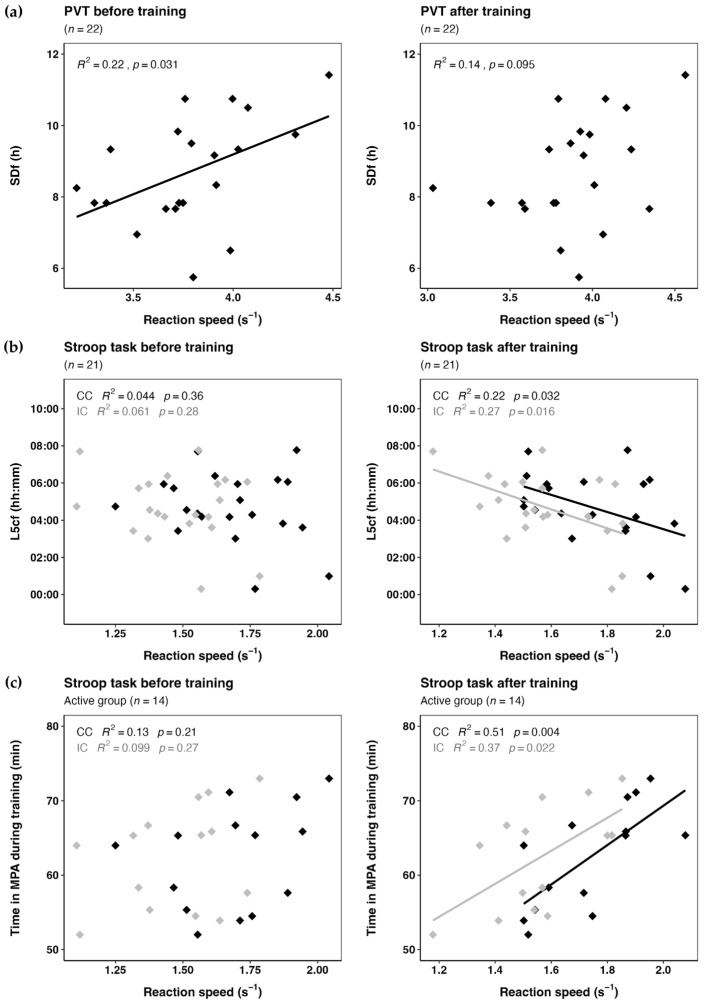
Effects of sleep, circadian phase, and time in MPA on attentional performance: (**a**) PVT before training vs. sleep duration on free days and PVT after training vs. sleep duration on free days for the whole sample, not significant after Benjamini–Hochberg correction; (**b**) Stroop task before training vs. L5c on free days and Stroop task after training vs. L5c on free days for the whole sample; (**c**) Stroop task before training vs. time in MPA during training and Stroop task after training vs. time in MPA during training for the more active group of dancers, significant after Benjamini–Hochberg correction. Abbreviations: CC, congruent condition; IC, incongruent condition; MPA, moderate physical activity; *p*, *p*-value.

We found a significant correlation between PVT performance before training and sleep duration on free days as estimated by MCTQ, but not after training (Figure 2a), nor with actimetry data, nor in training days. We found significant correlations between Stroop performance after training for both conditions and L5c on free days, but no correlation was found before training (Figure 2b). We did not find correlations between speed and time spent in MPA for the whole sample. When we examined the correlation between reaction speed and the exercise variable for the active group (*n* = 14), we found significant correlations between the time spent in MPA and Stroop performance after training for both congruent and incongruent conditions (Figure 2c).

We then estimated regression models to study the effects of both circadian (L5c on free days) and exercise (time spent in MPA) parameters on reaction speed after training in the active group of dancers (Table 4), including the correlation shown in Figure 2c. The best-fitting model revealed a significant effect of the circadian parameter for the incongruent condition, whereas the exercise parameter was significant for the congruent condition. In the less active group, the analysis did not yield significant results.

We did not find any correlations between performance measures and intradaily (IV), interdaily (IS), or weekly (SJL) circadian variability, nor between performance measures and individual variability in physical activity during training, assessed by intensity gradient and intensity intercept.

## 3. Discussion

### 3.1. Study Findings

Consistently with previous reports of changes in cognitive performance throughout the day [10], we confirmed that dancers trained in the morning had better cognitive performance after training (at noon) than before (early morning). Also as expected [36], we observed more pronounced effects in the task requiring greater cognitive effort (Stroop incongruent condition) than in simpler tasks such as the PVT and the Stroop congruent condition. As a novel contribution of this study on dancers with late chronotypes on average [32,35], we found that: (a) the longer the sleep on the free days, the better the cognitive performance (PVT) early in the morning; (b) the later the circadian phase (estimated by L5c), the worse the Stroop performance after training; and (c) the longer the MPA during training, but the not variability in individual exercise intensity, the better the Stroop performance after training.

The Uruguayan Dance School model has been extensively studied to compare circadian and sleep traits between the morning (08:30 to 12:30) and the night shift (20:00 to 24:00) [32,33,34,35]. In this study, we analyzed for the first time dancers’ cognitive performance. To do that, we focused on morning-shift dancers, who are challenged to train early in the morning despite having late chronotypes on average. The population of dancers assessed here showed the same characteristics previously reported for dancers trained in the morning shift by self reports and/or actigraphy [32,33]: (a) late chronotypes; (b) high SJL; (c) intermediate circadian preference; (d) later sleep timing in free days than in training days; and (e) no sleep deficit. Although both subjective (MSFsc) and objective (L5c on free days) proxies of circadian phase correlated as expected, we did not find significant differences in L5c between training and free days as previously reported in morning-shift dancers [33]. A possible explanation could be the changes observed in chronotype and circadian rhythms during the COVID pandemic [37,38]. During the first semester, these dancers attended remote classes, which probably influenced their sleep habits. Therefore, the 15-day period of actimetry measures, performed one month after resuming in-person training, may not have coincided with individual subjective assessment. Regarding circadian preference, the absence of correlation between MEQ score and MSFsc or L5cf reinforces how these widely used indicators reflect different circadian features. The impact of the strict weekly dance school schedule seems to be better captured by instruments that are more dynamic and sensitive to changes in environmental cues.

Both circadian rhythms and sleep homeostasis modulate cognitive performance [9,10]. While selective visual attention has been reported to be most strongly modulated by sleep homeostasis, inhibitory control appeared to be most strongly modulated by circadian phase [36]. The positive correlation between PVT performance at 08:30 and weekend sleep duration (Figure 2a) could be explained by the misalignment of circadian rhythms during the week, which likely led to accumulated fatigue. Those who managed to sleep longer on the weekend may have achieved more restorative rest, improving cognitive performance on vigilance tasks like the PVT. However, the significance did not remain after Benjamini–Hochberg correction. Our study did not find associations between PVT reaction speed and sleep duration estimated by accelerometry. This discrepancy between both instruments reflects the complexity of assessing circadian parameters in populations with weekly differences in sleep patterns and underscores the importance of using a comprehensive assessment approach. While questionnaires provide insights into sleep habits, actimetry could be thought of as a long-exposure photograph of sleep patterns within a limited time window. On the other hand, as we mentioned above, this specific population slept the recommended hours for the age, and, with the notable exception of SJL, did not exhibited fragmented or irregular sleep nor unbalanced rest–activity patterns as assessed by intradaily variability, interdaily stability, and relative amplitude. As participants were not sleep deprived, it is understandable that weak or nonsignificant effects on alertness were observed.

A challenging task demanding higher-order cognitive function, for example, response inhibition, is expected to be more susceptible to circadian modulation than to homeostatic pressure [36]. Regarding the attentional Stroop task, we found an association between circadian phase (estimated by L5c on free days) and reaction speed after training in both congruent and incongruent conditions, with faster reaction times observed in dancers with earlier circadian phases (Figure 2b). It is interesting to note that despite the inconsistencies observed in the actimetric data to evaluate sleep patterns, L5c on free days still stands as an objective proxy of circadian phase. Our results are in line with previous findings showing that cognitive performance earlier in the day was worse in late chronotypes than in early ones [15]. Surprisingly, no similar association was found before training; however, the heavily skewed chronotype distribution towards extreme eveningness in our sample may explain this lack of associations early in the morning, together with the difficulty of the Stroop task.

The short-term effects of exercise on cognitive performance have been extensively reported [23,39,40], but the effect of long-term training on attentional processes of young adults has been less explored [41,42]. Dancers are a great model to test this, since they train regularly and spend even more time in MPA and MVPA than the recommended for their age group [26]. We did not find the expected correlation between response speed and the time spent in MPA during training when we tested all the participants. However, dancers were not homogeneous regarding their activity levels but showed two clear profiles. The most active dancers showed a less negative intensity gradient, lower intercept gradient, and higher mean acceleration, considered as a better activity profile [43]. Among this group (Figure 2c), time spent in MPA was significantly associated with an improvement in attentional performance after training in both selective attention (congruent condition) and inhibition control (incongruent condition). Interestingly, our model revealed differences when both circadian and exercise parameters were considered. The circadian effect was more pronounced under the most challenging condition, while time spent in MPA had a greater impact under the easier condition. Although the interaction between the circadian phase proxy and time in moderate exercise was not statistically significant in the models we ran, it suggested a potential trend that warrants further exploration. The impairing effect of later circadian phases may have been counteracted by the positive effect of moderate exercise, although a larger sample would be needed to detect this effect with greater confidence. Nevertheless, despite the small sample size, our results align with previous research [22,25] and provide evidence that exercise can enhance cognitive performance in young adults with late chronotypes, particularly performing at challenging times of the day. Interestingly, none of the parameters that measure variability correlated with performance, nor were they different between active and light-mover dancers. Overall, these results reinforce the role of exercise and individual circadian phase in the causality of performance improvement, with varying effects depending on the cognitive demands of each task. Despite extensive research on the effect of exercise on cognition in athletes, there remains a significant gap in studies on young professional dancers from a chronobiological perspective. The ecological model of the Uruguayan National Dance School, in which dancers train in the morning despite their late chronotypes, helps bridge that gap.

### 3.2. Limitations

As mentioned before, the study was conducted during the COVID pandemic, which limited its design and scope. Tests were completed immediately before and after the training session in the dance school’s own facilities. These time and space constraints, inherent to the ecological nature of the model, led us to present abbreviated versions of the attentional tasks, which may have limited our ability to capture greater effects. Furthermore, because of these special conditions, we were unable to assess individual day-to-day variability in performance and preceding night’s sleep, which would give us deeper insights into circadian influence and the impact of sleep and physical activity on attention. However, we still consider that our results are reliable because of the regular schedule of the school and previous reports on the relative stability of cognitive speed across days [36]. Because of the anonymization process of the data, we could not assess the type of dancer (folkloric or contemporary) the participants were. We speculate that dance style contributed to distinct activity profiles. Ideally, we would have examined performance across a range of chronotypes at different times of day. However, in the context of this dance school, the participants were predominantly late chronotypes, and the goal was to assess their performance within their training natural environment. Finally, as we recruited a very specific group of trained dancers following a regular schedule, our findings may not be extrapolated to other populations. Future research should specify the type of dance practiced during the training sessions, increase the sample size, include a sedentary control group, consider day-to-day individual variability on performance along with information from the previous night’s sleep, and incorporate measures taken in the evening. Those conditions would allow a finer analysis, distinguishing more clearly the complexity of the effects outlined here.

## 4. Materials and Methods

### 4.1. Participants

In September–October 2021, twenty-two dancers (18 females, 18–28 years) attending the morning shift of the Uruguayan Public School for Professional Training in Contemporary and Folkloric Dance (Escuela Nacional de Formación Artística Sodre, ENFAS, Ministerio de Educación y Cultura, Uruguay) participated in this study. Participants were screened for prior diagnoses of psychiatric, neurological, or sleep disorders and medications known to affect sleep. All participants gave written informed consent. This study was evaluated by the Ethics Committee of the School of Psychology, Universidad de la República, and complied with the principles outlined by the Declaration of Helsinki [44].

### 4.2. Circadian and Sleep Habits Characterization by Self-Report

Circadian characterization was assessed using the Spanish version of the Munich Chronotype Questionnaire (MCTQ) [45]. The midsleep point on free days corrected for sleep debt on workdays (MSFsc) was used as a proxy of individual chronotype [46]. We calculated the social jetlag (SJL) as the absolute difference between the midpoints of sleep on training and free days [47], as a measure of weekly variation [48]. Circadian preference was assessed using the Spanish version of the Morningness–Eveningness Questionnaire (MEQ) [49].

### 4.3. Circadian and Physical Activity Objective Measures

Physical activity, circadian and sleep data were acquired using portable accelerometers (GENEActiv, Activinsights Ltd., Kimbolton, UK) on the nondominant wrist for 15 d with a sampling frequency of 10 Hz. Data were extracted with the GENEActiv software version 4.0.12 (Activinsights Ltd., Kimbolton, UK) and analyzed with the GGIR package [50]. As a circadian phase proxy, we examined the L5h center (the timing of the least active 5 hrs in a day) on free days. We estimated sleep onset, sleep end and sleep duration for both training and free days. Circadian rhythms parameters included interdaily stability (IS, ranging from 0 to 1, representing the degree of consistency of activity patterns from day to day), intradaily variability (IV, ranging from 0 to 2, with higher values indicating a more fragmented rhythms), and relative amplitude (RA, ranging from 0 to 1, reflecting the difference between the activity during the 10 most active hours, M10, and the activity during the 5 least active hours, L5), calculated as (M10 − L5)/(M10 + L5) [51,52]. Exercise characterization was performed based on accelerometer-derived movement behavior features from literature [43]. The PA intensity was categorized into three levels, light (LPA, PA < 93.2 mg), moderate (MPA, 93.2 mg < PA < 418.3 mg) and vigorous PA (VPA, PA ≥ 418.3 mg) [53]. We assessed time spent in MPA during the training window, from 08:30 to 12:29, Monday through Friday. We used average acceleration, intensity gradient, and intensity intercept to provide a complementary description of the dancers’ activity profiles, reflecting individual variance. The intensity gradient describes the intensity distribution over a period of time. A less negative value with a lower intercept reflects a better intensity profile [43]. After detecting a bimodal distribution in the time spent in MPA, we categorized the dancers into two groups, an active group and a less active group, using the mean as the cutoff.

### 4.4. Justification for the Use of Independent Variables Indicators

We selected the best suited indicators for the independent variables of which the influence was evaluated. Sleep duration was selected as a proxy of sleep homeostasis [10]. Circadian phase was assessed using two self-reported instruments (that delivered two indicators: chronotype by MSFsc and circadian preference by MEQ) and objective actimetry (L5cf) [54]. MEQ score is more reflective of a psychological trait than of actual behavior [13] and does not account for weekly differences. MSFsc is sensitive to environmental changes, is estimated in local time, and is ideal for application on structured schedules, such as our dance model. However, it excluded participants because of alarm use on free days. For these reasons, we selected L5cf as the most suitable indicator to assess the influence of the circadian phase on performance in this specific population. We selected time in MPA as an indicator to assess the potential effects of moderate-intensity exercise and the intensity gradient and intercept as indicators of individual exercise intensity variability [22,43]. We selected three indicators of individual variability in circadian rhythms to further analyze the influence of variability in three time scales: intradaily (IV), interdaily (IS), and weekly (SJL) [48,51].

### 4.5. Performance

We administered a 2 min version of the psychomotor vigilance task (PVT) to assess alertness [15] and a visual version of the Stroop Task (ST) to assess selective attention and inhibitory control, before and after the morning dance training [55]. The tasks were conducted only once at each time (before/after training) per participant, counterbalanced to avoid learning or order effect, within midweek. Participants were randomly assigned to have their first session at either 08:30 or 12:30, with each session lasting 10 min.

For the PVT task, a yellow ms counter was used as a stimulus. Participants were instructed to observe a red circle on the computer screen and to press the spacebar as soon as the counterstimulus appeared on the screen, which stopped the counter and displayed the reaction time in ms for a period of 1 s. The interstimulus interval, defined as the period between the last response and the appearance of the next stimulus, varied randomly between 2 and 10 s [56]. A 10 s prepractice test was performed. Reaction time (RT) in ms was recorded. A response was considered valid if the RT was ≥100 ms and <1000 ms. Reaction speed was taken as the dependent variable, obtained from the reciprocal transformation of RT (1/RT). To calculate the individual mean reaction rate, the transformed values were averaged [56,57].

For the Stroop task, words describing four colors (red, yellow, green, and blue) were presented in the same color that they described (CC, congruent condition) or in a nonmatching color (IC, incongruent condition). Participants were asked to press the key representing the color of the text, but not the meaning of the word. The interval between stimuli was fixed (1100 ms). The target stimulus remained on the screen for 2000 ms or until a response was registered. Four blocks of stimuli were presented with 32 trials each, 50% congruent, 50% incongruent (128 trials in total), and reaction times in ms (RT) were recorded. A response was regarded as valid if RT was ≥200 ms. Response speed was the dependent variable. To calculate the individual mean response speed, each RT (in seconds) was reciprocally transformed (1/RT), and the transformed values were then averaged [56,57]. Incorrect answers were discarded.

### 4.6. Data Analysis

We conducted all statistical analyses using the R statistical software version 4.4.1 (14 June 2024) [58] in the RStudio environment version 2024.12.0.467 [59]. We used paired *t*-tests to compare sleep parameters on free versus training days, and performance before and after the training session. We used the Wilcoxon rank sum test for nonparametric data. We measured effect size using Cohen’s d. We conducted regression models to estimate the associations between cognitive performance, circadian phase proxies, and exercise variables. We employed Benjamini–Hochberg correction to adjust for multiple comparisons. Values of *p* ≤ 0.05 were considered statistically significant.

## 5. Conclusions

Overall, our study expands the current understanding of the interplay among circadian rhythms, exercise, and their impact on cognition. Attentional performance is expected to improve after noon because of circadian rhythmicity, but our results also suggest different associations between sleep, circadian phase, and moderate exercise on tasks demanding varying degrees of difficulty. The less demanding task at the more challenging time was associated with sleep duration on free days. The more challenging task, which required inhibitory control, was associated with circadian phase at the most favorable time, whereas the task demanding selective attention was associated with time spent in moderate exercise during training, although only for more active dancers. These results reflect the delicate complexity of attentional processes and their modulation by both behavior and endogenous biological rhythms. Within the framework of this model, regular morning dance training could enhance cognitive performance, potentially counteracting the impairment typically associated with later chronotypes at a challenging time of day. Our findings could be used to inform the design of healthier training schedules for both dancers and athletes, taking into account time of day, sleep habits, and individual circadian phases.

## Figures and Tables

**Table 1:** Dancer’s profile.

**Demographic Data (*n* = 22)**	
Age	22.95 (3.27)
Sex (Female)	18 (82%)
**Questionnaire data (*n* = 22)**	
MSFsc (hh:mm, *n* = 15)	05:41 (01:20)
SJL (h, *n* = 22)	2.45 (1.26)
MEQ (*n* = 22)	48 (7)
**Accelerometer data (*n* = 21)**	
L5cf (hh:mm)	04:40 (01:52)
IS	0.49 (0.08)
IV	0.32 (0.06)
RA	0.87 (0.05)

Values are shown as mean (sd) or *n* (%). Abbreviations: MSFsc, midsleep point on free days corrected for sleep debt on workdays; SJL, social jetlag; MEQ, Morningness–Eveningness Questionnaire score; L5cf, least active five hours on free days; IS, interdaily stability; IV, intradaily variability (IV); RA, relative amplitude.

**Table 2:** Circadian parameters on training and free days.

	Variable	Free Days	Training Days	*t*	*p*-Value		Cohen’s d
MCTQ (*n* = 22)	Midsleep (hh:mm)	05:53 (01:22)	03:29 (00:35)	8.37	**3.97 × 10^−8^**	***	1.78 (large)
	Sleep-onset (hh:mm)	01:31 (01:38)	23:59 (00:53)	4.68	**1.27 × 10^−4^**	***	0.99 (large)
	Sleep-end (hh:mm)	10:15 (01:28)	06:58 (00:40)	9.66	**3.54 × 10^−9^**	***	2.06 (large)
	Sleep-duration (h)	8.73 (1.47)	6.98 (1.03)	5.18	**3.94 × 10^−5^**	***	1.1 (large)
Accelerometry (*n* = 21)	L5c (hh:mm)	04:40 (01:52)	04:20 (00:45)	1.03	3.13 × 10^−1^	(ns)	-
	Sleep-onset (hh:mm)	01:50 (01:18)	00:35 (00:45)	4.68	**1.44 × 10^−4^**	***	1.02 (large)
	Sleep-end (hh:mm)	09:16 (01:28)	08:01 (00:45)	4.39	**2.80 × 10^−4^**	***	0.96 (large)
	Sleep-duration (h)	7.43 (1.05)	7.43 (0.71)	0.05	9.60 × 10^−1^	(ns)	-

Values are shown as mean (sd). The significance is shown with paired two-sample *t* tests. Statistically significant tests (*p* < 0.05) are shown in bold. Significant after Benjamini–Hochberg correction. Significance is presented as *** = *p* < 0.001; ns = not significant.

**Table 3:** Performance before and after morning training sessions.

Task	Before Training	After Training	*t*	*p*-Value	Cohen’s d
PVT (s^−1^)	3.77 (0.312)	3.89 (0.325)	**−2.31**	**0.031**	−0.49 (small)
Stroop CC (s^−1^)	1.66 (0.216)	1.74 (0.191)	**−2.24**	**0.036**	−0.48 (small)
Stroop IC (s^−1^)	1.46 (0.198)	1.57 (0.19)	**−2.94**	**0.008**	−0.63 (moderate)

Values are shown as mean (sd). Significance is shown with paired two-sample *t* tests. Statistically significant tests (*p* < 0.05) are shown in bold. Abbreviations: CC, congruent condition; IC, incongruent condition. Significant after Benjamini–Hochberg correction.

**Table 4:** Influence of circadian phase and time in moderate exercise on attention.

	Stroop IC After Training—Active Group				Stroop CC After Training—Active Group			
	b ± SE	b ± SE	b ± SE	b ± SE	b ± SE	b ± SE	b ± SE	b ± SE
(Intercept)	1.83 *** (0.09)	0.53 (0.39)	1.12 ** (0.42)	1.87 * (1.08)	1.97 *** (0.10)	0.53 * (0.34)	0.91 (0.41)	1.20 (1.06)
L5cf	−0.06 * **(0.02)**		−0.05 * **(0.02)**	−0.19 (0.19)	−0.05 * **(0.02)**		−0.03 (0.02)	−0.08 (0.19)
Time in MPA		0.02 * **(0.01)**	0.01 (0.01)	−0.00 (0.02)		0.02 ** **(0.01)**	0.02 * **(0.01)**	0.02 (0.01)
L5cf: Time in MPA				0.00 (0.00)				0.00 (0.00)
Observations	14	14	14	14	14	14	14	14
R^2^/R^2^ adjusted	0.47/0.42	0.37/0.32	0.58/0.50	0.60/0.48	0.34/0.29	0.51/0.47	0.6/0.53	0.60/0.48

Linear regression models. Significance is presented as * = *p* < 0.05, ** = *p* < 0.01, *** = *p* < 0.001. Abbreviations: CC, congruent condition; IC, incongruent condition; MPA, moderate physical activity. Significant after Benjamini–Hochberg correction, except for the fifth model from left.

## Data Availability

The original data presented in the study are openly available in OSF at https://osf.io/832cs.

## Abbreviations

The following abbreviations are used in this manuscript:

ENFAS	Escuela Nacional de Formación Artística del SODRE
IS	Interdaily stability
IV	Intraday variability
MCTQ	Munich Chronotype Questionnaire
MEQ	Morningness–Eveningness Questionnaire
MPA	Moderate physical activity
MVPA	Moderate to vigorous physical activity
MSFsc	Midsleep point on free days corrected for sleep debt on workdays
PVT	Psychomotor vigilance task
RA	Relative amplitude
